# Knowledge translation research: Teaching psychopharmacology using research domain criteria

**DOI:** 10.1192/j.eurpsy.2021.1588

**Published:** 2021-08-13

**Authors:** L. Mehl-Madrona, B. Mainguy

**Affiliations:** 1 Medical Arts And Humanities Program, University of Maine, Orono, United States of America; 2 Education Division, Coyote Institute - Canada, Ottawa, Canada

**Keywords:** Psychiatric diagnosis, Research Domain Criteria, Psychopharmacology, Neural circuitry

## Abstract

**Introduction:**

Research Domain Criteria are coming to be required for applications for mental health research funding in the United States.

**Objectives:**

To translate contemporary neuroscience research into teaching medical residents how to prescribe psychiatric medications.

**Methods:**

We explore the neuroscience literature regarding neural circuitry and psychiatric symptoms and examine the neurotransmitters associated with those circuits. We associate psychiatric symptoms with the neural circuitry that produces those symptoms. We correlate medications with circuits which they might affect and symptoms they might ameliorate.

**Results:**

RDC is an alternative to DSM and ICD-10. Contemporary scientific diagnoses are not based on neuroscience. They are overlapping, contradictory, often vague, and hinder adequate research. Diagnoses are needed that are based on brain circuitry and function rather than ”expert” opinion. The basis for RDC lies in psychiatric disorders being brain disorders with a primary focus on circuitry function. This contrasts with neurological disorders that have identifiable structural lesions. Symptoms are normally distributed and exist in everyone. RDC proposes to seek the distribution of traits and characteristics, defining abnormal as the extremes of these distributions rather than by defining mental disorders by signs and symptoms which give a diagnosis. We ask what are the brain system that primarily implements the traits, functions, and characteristics of interest. We explore what accounts for the development of dysregulation or dysfunction in these systems alongside normal-to-abnormal dimensions? We describe resident reactions to this style of teaching and show greater comfort in prescribing medications.

**Conclusions:**

Translating Research Domain Criteria into psychiatric prescribing will move psychopharmacology into contemporary neuroscience.

